# Renal Effects of Angiotensin-Converting Enzyme Inhibitors and Angiotensin Receptor Blockers in Patients with Liver Cirrhosis: A Nationwide Cohort Study

**DOI:** 10.1155/2019/1743290

**Published:** 2019-10-10

**Authors:** Wei-Fan Hsu, Shi-Hang Yu, Jaw-Town Lin, Jaw-Ching Wu, Ming-Chih Hou, Yi-Hsiang Huang, Chun-Ying Wu, Cheng-Yuan Peng

**Affiliations:** ^1^Division of Hepatogastroenterology, Department of Internal Medicine, China Medical University Hospital, Taichung, Taiwan; ^2^Graduate Institute of Biomedical Science, China Medical University, Taichung, Taiwan; ^3^Division of Translational Research, Taipei Veterans General Hospital, Taipei, Taiwan; ^4^Digestive Medicine Center, China Medical University Hospital, Taichung, Taiwan; ^5^Institute of Clinical Medicine, National Yang-Ming University, Taipei, Taiwan; ^6^Department of Medical Research, Taipei Veterans General Hospital, Taipei, Taiwan; ^7^Department of Internal Medicine, Taipei Veterans General Hospital, Taipei, Taiwan; ^8^Division of Gastroenterology & Hepatology, Taipei Veterans General Hospital, Taipei, Taiwan; ^9^Department of Public Health, China Medical University, Taichung, Taiwan; ^10^School of Medicine, China Medical University, Taichung, Taiwan

## Abstract

**Background:**

The use of angiotensin-converting enzyme inhibitors (ACEis) and angiotensin receptor blockers (ARBs) carries a risk of renal function deterioration in cirrhotic patients with ascites. However, whether the long-term use of ACEis/ARBs is safe in cirrhotic patients without ascites remains unknown.

**Methods:**

In this nationwide cohort study, we identified 311,361 newly diagnosed cirrhotic patients between January 1997 and December 2013. To avoid indication and immortal time biases, patients receiving regular ACEi/ARB therapy, defined as the ACEi/ARB cohort, were matched to patients receiving regular calcium channel blockers (CCBs), defined as the CCB cohort, at a ratio of 1 : 1 by age, sex, and propensity scores for comorbidities and medications (2,188 patients in each cohort). Cumulative incidence rates and multivariate analyses of end-stage renal disease (ESRD) risk were adjusted for competing mortality.

**Results:**

The 10-year cumulative incidence rates of ESRD were 2.32% (95% confidence interval [CI]: 1.45–3.20) in the ACEi/ARB cohort and 1.70% (95% CI: 1.03–2.36) in the CCB cohort (*P* = 0.610). In multivariate analyses, ACEi/ARB use was not associated with a higher risk of ESRD in cirrhotic patients (hazard ratio [HR] = 1.15; 95% CI: 0.69–1.94, *P* = 0.591). In the sensitivity test, the 10-year cumulative incidence rates of ESRD in cirrhotic patients with ascites were 6.50% (95% CI: 0.54–12.46) and 1.24% (95% CI: 0.00–2.71) in ACEi/ARB and CCB cohorts, respectively (*P* = 0.090).

**Conclusions:**

Long-term ACEi/ARB use was not associated with a higher risk of ESRD in cirrhotic patients. However, the risk of ESRD tended to increase in cirrhotic patients with ascites.

## 1. Introduction

Portal hypertension is the main complication and prognostic marker of liver cirrhosis, and it results in gastroesophageal varices, hepatic encephalopathy, and ascites [1]. Nonselective *β*-blockers (BBs) are the standard medications for portal hypertension [2], but 15% of patients taking BBs experience intolerable side effects, and less than 40% achieve therapeutic goals [3]. Identification of alternative medications for portal hypertension is important. In 1999, Schneider et al. [4] showed that losartan, an angiotensin receptor blocker (ARB), significantly lowered portal pressure in patients with liver cirrhosis, but subsequent clinical studies have revealed the opposite. Studies have found that irbesartan or losartan was not more effective in lowering portal pressure than BBs but, in fact, deteriorated renal function in patients with liver cirrhosis [5, 6]. Renal failure in patients with liver cirrhosis has become a concern [7]. The American Association for the Study of Liver Diseases (AASLD) cautions that use of angiotensin-converting enzyme inhibitors (ACEis) and ARBs in cirrhotic patients with ascites may be harmful [8]. The European Association for the Study of the Liver (EASL) also states that ACEis and ARBs should generally not be used in (cirrhotic) patients with ascites [9]. Angiotensin is a vasoconstrictor that counters the vasodilatory effect of nitric oxide in splanchnic circulation [7, 8], and ACEis and ARBs inhibit the effects of angiotensin, which are expected to lower blood pressure and deteriorate renal function [8, 9]. However, evidence from large-scale studies on the safety of long-term ACEis/ARBs use in patients with liver cirrhosis is lacking.

Several large-scale, randomized, placebo-controlled clinical trials have discovered that ACEis and ARBs have renoprotective effects in diabetic [10, 11] and nondiabetic patients with nephropathy [12, 13] and in patients at high vascular risk (aged ≥ 55 years with established atherosclerosis or diabetes with end-organ damage) [14, 15]. However, their role in patients with liver cirrhosis remains unclear.

In this population-based nationwide cohort study, we used the Taiwan National Health Insurance Research Database (NHIRD) and the Registry for Catastrophic Illness Patient Database (RCIPD), a subsystem of the NHIRD, to investigate the renal effects of long-term ACEis/ARBs use in patients with liver cirrhosis.

## 2. Methods

### 2.1. Study Population

The data of this population-based cohort study were derived from the NHIRD, which contains prospectively collected nationwide health-care data, including demographic data, all records of outpatients' visits and hospitalizations, details of prescriptions, operation codes, expenditure amounts, and diagnostic codes according to the International Classification of Disease, Ninth Revision, Clinical Modification (ICD-9-CM) from January 1, 1997, to December 31, 2013 [16]. Because it is a single and universal welfare insurance system, the Taiwan National Health Insurance program covers more than 99% of the entire population of 23.53 million in Taiwan [17–20]. This study was approved by the Research Ethics Committee of the National Health Research Institute in Taiwan and the Institutional Review Board of China Medical University Hospital (Certification Number: CMUH104-REC2-115). The identification number of each patient was encrypted for privacy protection; thus, the need for informed consent was waived.

### 2.2. Definition of Study Cohorts

Identification of patients with liver cirrhosis was based on specific codes (571.2, 571.5, and 571.6) once at admission or more than three times at the outpatient clinic. We also identified cirrhotic patients with ascites based on specific codes of liver cirrhosis and ascites (789.5) or a reimbursement code for ascites analysis (16002C). This study enrolled patients with liver cirrhosis who had continuously used hypotensive regimens of ACEi/ARB or calcium channel blockers (CCBs) for more than 63 days in the first 90 days. To avoid indication and immortal time biases, patients who continuously used ACEi or ARB (defined as the ACEi/ARB cohort) were randomly matched with those continuously using CCB (defined as the CCB cohort) at a ratio of 1 : 1. To optimize comparability among the study cohorts, patients were not enrolled if they had cancer, as identified in the RCIPD, before the index date [17–20]. The detailed regimens of ACEi/ARB and CCB are shown in [Supplementary-material supplementary-material-1].

Patients who attained major outcomes before enrolment or the index date were excluded. Those who used ACEi/ARB and CCB at the same time or these two kinds of drugs in any sequential orders for more than 30 days per year were also excluded.

### 2.3. Major Outcome Measurements

All patients were followed up for the occurrence of the outcomes from the last day of ACEi/ARB or CCB administration in the first 90 days to death or December 31, 2013, whichever came first. The major outcome was end-stage renal disease (ESRD). ESRD was defined as irreversible renal failure requiring long-term dialysis and was ascertained by the certification in RCIPD [20].

### 2.4. Adjustment for Confounding Factors

The use of certain medications, including nonsteroidal anti-inflammatory drugs (NSAIDs) or cyclooxygenase-2 inhibitors (COX-2), aspirin, statins, metformin, and BBs, which may influence renal function, was analyzed. Drug users were defined as patients who used more than one tablet per month during the study period. The propensity score was measured using logistic regression analysis consisting of demographic factors including age, sex, comorbidities, and concomitant medications. Comorbidities, identified based on ICD-9-CM codes, included viral hepatitis B (070.2, 070.3, and V02.61), viral hepatitis C (070.41, 070.44, 070.51, 070.54, 070.70, 070.71, and V02.62), alcoholic liver disease (571.0–571.3), other chronic hepatitis (571.40, 571.41, and 571.49), hypertension (401–405, A260, and A269), diabetes mellitus (249–250), congestive heart failure (428), and hyperlipidemia (272) ([Supplementary-material supplementary-material-1]).

Because death for patients with liver cirrhosis led us to apply informative censoring when calculating the major outcome, mortality in enrolled patients was regarded as a competing risk event and was adjusted for through competing risk analyses.

### 2.5. Statistical Analysis

Continuous variables are reported as median (25%–75% interquartile range) and categorical variables as number (percentage). The modified Kaplan–Meier method and Gray's method were used to calculate and compare the cumulative incidence rates of ESRD [21]. After matching the propensity score of comorbidities and medications, multivariate analyses were conducted using Cox proportional hazard model to estimate the hazard ratio (HR) and 95% confidence interval (95% CI) for examining the independent association of ACEi/ARB and CCB with major outcomes. All analyses were performed using SAS software (version 9.4, SAS Institute Inc., Cary, NC). The hazard ratio of the cumulative incidence in the competing risk analysis was calculated using R software with the “cmprsk_2.1-4” package (https://cran.r-project.org/src/contrib/Archive/cmprsk/). All reported *P* values were obtained from two-sided tests. Statistical significance was set at *P* < 0.05.

## 3. Results

### 3.1. Baseline Characteristics of the Study Population

**Figure 1:**
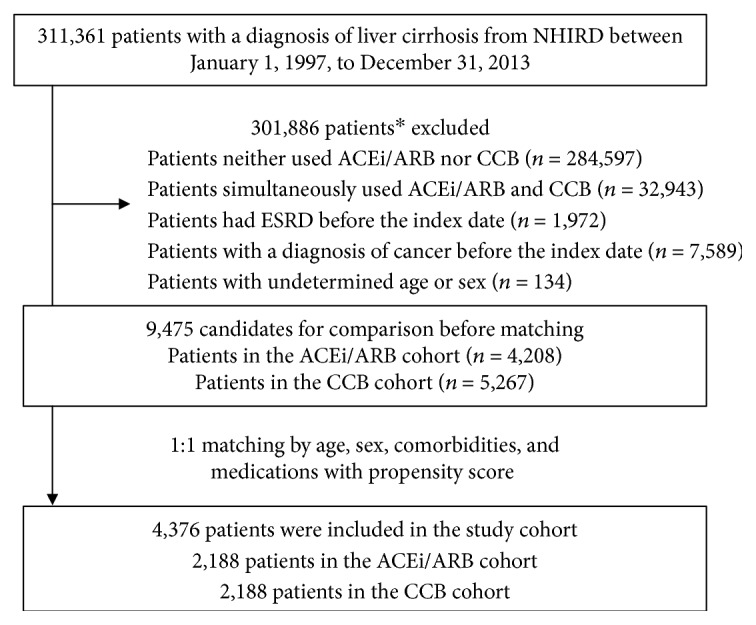
Flowchart of the enrollment process for patients with liver cirrhosis. ^∗^More than one exclusion criteria could overlap in a patient.

Between January 1, 1997, and December 31, 2013, we identified 311,361 newly diagnosed cirrhotic patients. We excluded 284,597 patients who used neither ACEi/ARB nor CCB for more than 63 days in the first 90 days; 32,943 who simultaneously used ACEi/ARB and CCB for more than 30 days per year; 1,972 who had ESRD before the index date; 7,589 who had cancer before the index date; and 134 without records of sex or age. Of note, more than one exclusion criteria could overlap in a patient. Finally, 9,475 cirrhotic patients (4,208 in the ACEi/ARB cohort and 5,267 in the CCB cohort) were enrolled. After propensity score matching of patients in the two cohorts who showed no differences in demographic factors, viral hepatitis B, viral hepatitis C, hypertension, diabetes mellitus, congestive heart failure, hyperlipidemia, and concomitant use of BBs, statin, metformin, aspirin, NSAID, and COX-2, 4,376 patients (2,188 patients in each cohort) were eligible for comparison (Figure 1).

### 3.2. Cumulative Incidence Rates of ESRD in Patients with Liver Cirrhosis in the ACEi/ARB and CCB Cohorts

The median patient age was 67.75 (58.41–75.52) years in the ACEi/ARB cohort and 67.97 (58.58–75.30) years in the CCB cohort (*P* = 0.914). The median follow-up period in ACEi/ARB and CCB cohorts was 2.95 (1.26–5.78) and 3.14 (1.24–6.19) years (*P* = 0.089), respectively, with the longest observation period being 17 years (from January 1, 1997, to December 31, 2013) (Table 1).

**Figure 2:**
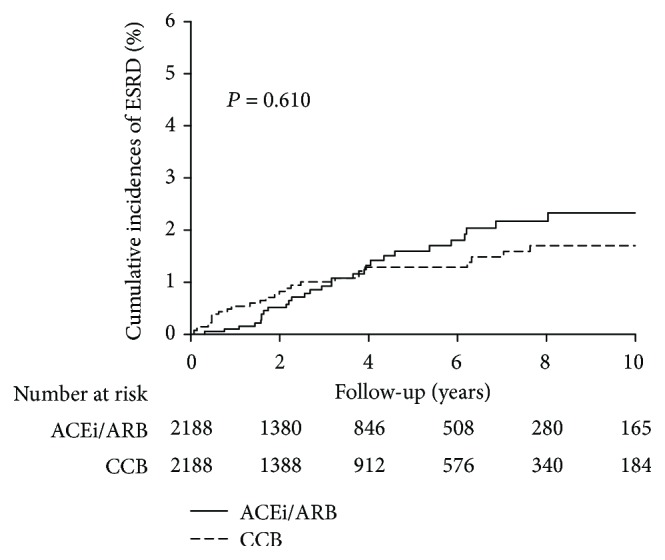
Cumulative incidence of ESRD in patients with liver cirrhosis that was analyzed using the modified log-rank test with death adjusted as a competing risk event.

Using the modified Kaplan–Meier method and Gray's method, the 10-year cumulative incidence rates of ESRD were 2.32% (95% CI: 1.45–3.20) and 1.70% (95% CI: 1.03–2.36) in the ACEi/ARB and CCB cohorts after adjustment for competing mortality (*P* = 0.610) (Figure 2).

### 3.3. Relative Risks of ESRD and Multivariate Stratified Analysis

Table 2 presents the Cox multivariate proportional hazard analysis for determining independent prognostic factors for ESRD.

**Figure 3:**
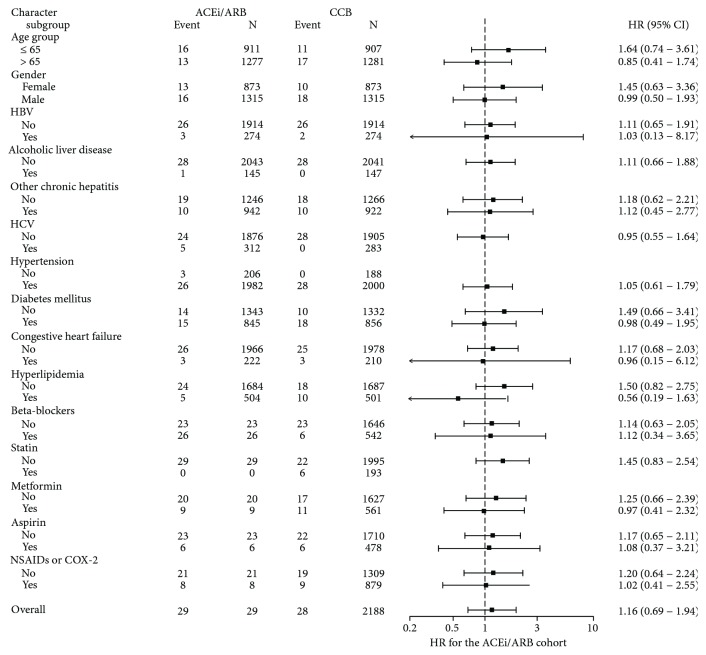
Multivariate stratified analyses for the association between ACEi/ARB or CCB therapy and ESRD risk in patients with liver cirrhosis. ACEi: angiotensin-converting enzyme inhibitor; ARB: angiotensin receptor blocker; COX-2: cyclooxygenase-2 inhibitor; CI: confidence interval; HR: hazard ratio; HBV: patients with hepatitis B virus infection; HCV: patients with hepatitis C virus infection; NSAID: nonsteroidal anti-inflammatory drug.

Enrolled patients who received ACEis/ARBs did not exhibit an increased risk of ESRD compared with patients who received CCB (HR = 1.15, 95% CI: 0.69–1.94; *P* = 0.591). Diabetes mellitus was an independent risk factor for ESRD (HR = 2.49, 95% CI: 1.29–4.82; *P* = 0.007). Patients who were younger (HR = 0.98, 95% CI: 0.96–1.00, *P* = 0.040) and patients who used NSAID or COX-2 exhibited a decreased risk of ESRD (HR = 0.51, 95% CI: 0.29–0.91; *P* = 0.022). Multivariate stratified analysis of all subgroups of patients was performed, and in the ACEi/ARB and CCB cohorts, the 10-year cumulative incidence rates of ESRD were not different across subgroups (Figure 3).

### 3.4. Cumulative Incidence Rates of ESRD in Cirrhotic Patients with Ascites in ACEi/ARB and CCB Cohorts

We also identified a subgroup of cirrhotic patients with ascites (*n* = 1,248; 540 in the ACEi/ARB cohort and 708 in the CCB cohort) who met the inclusion criteria. After matching at a 1 : 1 ratio by age, sex, and propensity scores for comorbidities and medications, 712 patients (356 patients in each cohort) were eligible for comparison ([Supplementary-material supplementary-material-1]).

The median age was 69.93 (57.31–78.38) years in the ACEi/ARB cohort and 69.68 (58.49–78.44) years in the CCB cohort (*P* = 0.891). The median follow-up period in ACEi/ARB and CCB cohorts was 1.67 (0.64–3.50) and 1.61 (0.64–3.49) years (*P* = 0.748), respectively ([Supplementary-material supplementary-material-1]). Using the modified Kaplan–Meier method and Gray's method, the 10-year cumulative incidence rates of ESRD were 6.50% (95% CI: 0.54–12.46) and 1.24% (95% CI: 0.00–2.71) in the ACEi/ARB and CCB cohorts after adjustment for competing mortality (*P* = 0.090) ([Supplementary-material supplementary-material-1]). No patient with hepatitis C virus infection or statin use developed ESRD in the CCB cohort; these two factors were excluded from Cox multivariate analysis. In Cox multivariate proportional hazard analysis, diabetes mellitus was still an independent risk factor for ESRD (HR = 6.66, 95% CI: 1.32–33.63; *P* = 0.022) in cirrhotic patients with ascites. Male patients (HR = 0.24, 95% CI: 0.06–0.96; *P* = 0.044) and younger patients (HR = 0.93, 95% CI: 0.90–0.97; *P* < 0.001) exhibited a decreased risk of ESRD ([Supplementary-material supplementary-material-1]).

We also identified a subgroup of patients with decompensated liver cirrhosis (*n* = 906, 453 patients in each cohort) after matching at a 1 : 1 ratio by age, sex, and propensity score for comorbidities and medications ([Supplementary-material supplementary-material-1]). Patients with decompensated liver cirrhosis were identified by repeated episodes of hepatic encephalopathy or gastroesophageal variceal bleeding or refractory ascites according to the liver cirrhosis-related catastrophic illness ([Supplementary-material supplementary-material-1]). Using the modified Kaplan–Meier method and Gray's method, the 10-year cumulative incidence rates of ESRD were 3.54% (95% CI: 1.49–5.60) and 2.17% (95% CI: 0.65–3.68) in the ACEi/ARB and CCB cohorts, respectively, after adjustment for competing mortality (*P* = 0.332) ([Supplementary-material supplementary-material-1]). Repeated episodes of hepatic encephalopathy or gastroesophageal variceal bleeding may not be directly related to renal function deterioration, and the trends for incidence rates of ESRD in patient with decompensated liver cirrhosis were similar to that in patients with liver cirrhosis.

## 4. Discussion

This population-based nationwide cohort study is the first study to investigate the renal effects of ACEis and ARBs in patients with liver cirrhosis. The results revealed that long-term ACEis/ARBs use was safe in cirrhotic patients without ascites. However, ACEis and ARBs potentially but nonsignificantly (*P* = 0.090) increased the risk of ESRD in cirrhotic patients with ascites. The 10-year cumulative incidence rates of ESRD were more than five times higher in cirrhotic patients with ascites taking ACEis or ARBs than in those taking CCBs. Our result supports the guidelines of AASLD and EASL [8, 9].

Managing renal failure in patients with liver cirrhosis is challenging [7], and several diagnostic criteria for kidney dysfunction in patients with liver cirrhosis have been proposed [22, 23]. Of note, the International Club of Ascites proposed new definitions of acute kidney injury (AKI) in patients with liver cirrhosis and recommended guidelines for management strategies for AKI in patients with liver cirrhosis [24]. AKI is also a powerful predictor of death in patients with liver cirrhosis [25]. In patients with advanced liver cirrhosis, arterial pressure is maintained by multiple vasoconstrictive systems, including the renin–angiotensin–aldosterone system and the sympathetic nervous system, and the antidiuretic hormone [7–9]. ACEis and ARBs inhibit the effects of angiotensin, which are expected to lower blood pressure and deteriorate renal function [8, 9]. However, evidence from large-scale clinical studies is lacking.

Randomized controlled trials have shown that ACEis/ARBs are renoprotective [10–15], but these trials have not addressed the effects of ACEis/ARBs on renal function in patients with liver cirrhosis, which have been suggested to have harmful effects by the AASLD and EASL [8, 9]. A study in Taiwan that used data from the NHIRD showed that losartan and ramipril reduced the incidence of ESRD in patients with chronic kidney disease, but the subgroup of patients with liver cirrhosis was not investigated [26]. It is unethical to design a study that investigates adverse effects in patients to challenge the guidelines. Therefore, we used the NHIRD to investigate the renal effects of ACEis/ARBs in patients with liver cirrhosis. Patients who took antihypertensive medications other than ACEis/ARBs were heterogeneous, and patients who needed multiple antihypertensives to control their blood pressure may exhibit several comorbidities, confounding our analysis. Randomized controlled trials have used amlodipine [10] or felodipine [13] users as the control group to investigate the renoprotective effect of ACEi/ARB. Thus, we chose cirrhotic patients taking CCBs as the matched cohort.

Diabetes mellitus is a well-known risk factor for ESRD [10, 11, 14, 15] and is an independent prognostic factor for poor survival and major complications of cirrhosis [27]. Our results also showed that diabetes mellitus was a risk factor for ESRD in patients with liver cirrhosis (HR = 2.49, 95% CI: 1.29–4.82) and in cirrhotic patients with ascites (HR = 6.66, 95% CI: 1.32–33.63). Furthermore, NSAIDs should be avoided in patients with liver cirrhosis because renal failure is more likely in these patients [7, 24, 28], and epidemiological data showed that NSAID use was a risk factor for ESRD in the Chinese population [29]. Therefore, our finding that NSAID or COX-2 decreased the risk of ESRD in cirrhotic patients (HR = 0.51, 95% CI: 0.29–0.91) is an example of confounding by contraindication [30]. Prior evidence has shown that statins and BBs are important medications affecting the survival or decompensation in patients with liver cirrhosis [28, 31–33]. In the present study, we performed propensity score matching of ACEi/ARB and CCB cohorts including patients taking these two types of medications. In stratified analysis, the 10-year cumulative incidence rates of ESRD were not different in the two cohorts across all subgroups, including subgroups stratified by age, sex, different liver diseases, comorbidities, and frequently confounding medications.

Schepke et al. [5] and Gonzalez-Abraldes et al. [6] have shown that irbesartan and losartan reduced the glomerular filtration rate in patients with liver cirrhosis 1 and 6 weeks after drug initiation, but the studies have enrolled only 13 and 18 patients using irbesartan and placebo and 23 and 14 patients using losartan and propranolol, respectively. Tandon et al. [34] summarized that regarding reducing the hepatic venous pressure gradient, the reduction ability of ACEis/ARBs was not significantly different from that of BBs, and ACEis/ARBs may have adverse effects on renal insufficiency in patients with decompensated liver cirrhosis [6]. However, their systemic review only included 213 patients taking ACEi/ARB and 186 patients taking placebo or BBs [34]. Our study included a larger sample, that is 4,376 patients (2,188 patients in each cohort), and compared the 10-year cumulative incidence rates of ESRD in patients with liver cirrhosis taking ACEis/ARBs or CCBs.

This study had several limitations. First, dynamic changes in renal function were unknown. Although only a few patients showed ESRD occurrence, which was the major outcome in this study, ESRD was a definite diagnosis in comparison with reversible chronic kidney disease. Furthermore, it is unethical to design a prospective study to investigate the side effects of ACEis/ARBs in cirrhotic patients. Second, although proteinuria is a key factor predicting renal function deterioration [35, 36], we could not correctly identify patients with proteinuria using the NHIRD. Before propensity score matching, patients in the ACEi/ARB cohort had a higher incidence of several comorbidities than those in the CCB cohort, including chronic hepatitis B (15.8% vs. 13.0%, *P* < 0.001), diabetes mellitus (47.9% vs. 33.0%, *P* < 0.001), congestive heart failure (24.4% vs. 8.2%, *P* < 0.001), and hyperlipidemia (31.8% vs. 21.5%, *P* < 0.001), and patients in the ACEi/ARB cohort also had higher statin (17.6% vs. 9.2%, *P* < 0.001) and aspirin use (30.1% vs. 22.2%, *P* < 0.001) ([Supplementary-material supplementary-material-1]). We reasonably speculate that physicians prescribe ACEi/ARB more frequently to prevent renal function deterioration in patients with proteinuria. Notably, the incidence of ESRD was not higher in the ACEi/ARB cohort before matching ([Supplementary-material supplementary-material-1]). Third, the subgroup of cirrhotic patients with ascites had a small sample size (356 patients in each cohort), which precluded the difference in the 10-year cumulative incidence rate of ESRD from reaching statistical significance. Nonetheless, the incidence of ESRD was more than five times higher in cirrhotic patients with ascites taking ACEis/ARBs than in those taking CCBs. Lastly, this nationwide cohort may only serve to address the renal effects of ACEi and ARB in cirrhotic patients, which only represents a small proportion of cirrhotic patients.

## 5. Conclusions

In conclusion, long-term ACEis/ARBs use did not increase the risk of ESRD in patients with liver cirrhosis, but they tended to increase the risk of ESRD in cirrhotic patients with ascites. ACEis and ARBs should be used with caution in cirrhotic patients with ascites, and future multicenter retrospective studies should collect detailed information on hepatic and renal biochemistries of cirrhotic patients taking ACEis or ARBs.

## Figures and Tables

**Table 1:** Baseline characteristics of study patients.

Characteristics	ACEi/ARB (*n* = 2,188)	CCB (*n* = 2,188)	
	*n* (%)	*n* (%)	*P* value
Age, y, median (IQR)	67.75 (58.41–75.52)	67.97 (58.58–75.30)	0.914
Gender			>0.999
Female	873 (39.9)	873 (39.9)	
Male	1315 (60.1)	1315 (60.1)	
Cause of cirrhosis			
Hepatitis B virus infection	274 (12.5)	274 (12.5)	>0.999
Hepatitis C virus infection	312 (14.3)	283 (12.9)	0.217
Alcoholic liver disease	145 (6.6)	147 (6.7)	0.952
Other chronic hepatitis	942 (43.1)	922 (42.1)	0.561
Comorbidity			
Hypertension	1982 (90.6)	2000 (91.4)	0.369
Diabetes mellitus	845 (38.6)	856 (39.1)	0.756
Congestive heart failure	222 (10.2)	210 (9.6)	0.577
Hyperlipidemia	504 (23.0)	501 (22.9)	0.943
Drug exposure			
Beta-blockers	528 (24.1)	542 (24.8)	0.648
Statin	159 (7.3)	193 (8.8)	0.067
Metformin	542 (24.8)	561 (25.7)	0.531
Aspirin	464 (21.2)	478 (21.9)	0.633
NSAIDs or COX-2	862 (39.4)	879 (40.2)	0.621
ESRD	29 (1.3)	28 (1.3)	>0.999
Competing mortality	739 (33.8)	928 (42.4)	<0.001
Follow-up year (IQR)	2.95 (1.26–5.78)	3.14 (1.24–6.19)	0.089

ACEi: angiotensin-converting enzyme inhibitor; ARB: angiotensin receptor blocker; CCB: calcium channel blocker; COX-2: cyclooxygenase-2 inhibitors; ESRD: end-stage renal disease; IQR: interquartile range; NSAIDs: nonsteroidal anti-inflammatory drugs.

**Table 2:** Multivariate Cox proportional hazards model analysis of risk of ESRD after adjustment for competing mortality.

	HR (95% CI)	*P* value
ACEi/ARB vs. CCB users	1.15 (0.69–1.94)	0.591
Age	0.98 (0.96–1.00)	0.040
Male vs. female	1.02 (0.59–1.80)	0.932
Hepatitis B virus infection	0.71 (0.28–1.82)	0.481
Hepatitis C virus infection	0.73 (0.28–1.90)	0.522
Alcoholic liver disease	0.20 (0.03–1.40)	0.104
Other chronic hepatitis	0.73 (0.42–1.27)	0.267
Hypertension	1.82 (0.57–5.74)	0.309
Diabetes mellitus	2.49 (1.29–4.82)	0.007
Congestive heart failure	1.31 (0.53–3.27)	0.559
Hyperlipidemia	1.25 (0.69–2.25)	0.465
Beta-blockers	0.71 (0.38–1.32)	0.278
Statin	1.14 (0.50–2.61)	0.760
Metformin	0.86 (0.44–1.68)	0.664
Aspirin	0.94 (0.49–1.81)	0.858
NSAIDs or COX-2	0.51 (0.29–0.91)	0.022

ACEi: angiotensin-converting enzyme inhibitor; ARB: angiotensin receptor blocker; CCB: calcium channel blocker; CI: confidence interval; COX-2: cyclooxygenase-2 inhibitor; ESRD: end-stage renal disease; HR: hazard ratio; NSAIDs: nonsteroidal anti-inflammatory drugs.

## Data Availability

The data used to support the findings of this study may be released upon application to the Bureau of National Health Insurance, Department of Health, which can be contacted at https://nhird.nhri.org.tw/en/.

## Supplementary Materials

Supplementary Materials Table S1: antihypertensive agents. Table S2: ICD-9-CM codes for the diagnoses of comorbidities. Table S3: baseline characteristics of cirrhotic patients with ascites. Table S4: multivariate Cox proportional hazards model analysis of risk of ESRD in cirrhotic patients with ascites after adjustment for competing mortality. Table S5: baseline characteristics of the patients with decompensated liver cirrhosis. Table S6: baseline characteristics of patients with liver cirrhosis taking ACEi/ARB or CCB before propensity score matching. Figure S1: flowchart of the enrollment process for cirrhotic patients with ascites. ^∗^More than one exclusion criteria could overlap in a patient. Figure S2: cumulative incidence of ESRD in cirrhotic patients with ascites that was analyzed using the modified log-rank test with death adjusted as a competing risk event. Figure S3: flowchart of the enrollment process for patients with decompensated liver cirrhosis. ^∗^More than one exclusion criteria could overlap in a patient. Figure S4: cumulative incidence of ESRD in patients with decompensated liver cirrhosis that was analyzed using the modified log-rank test with death adjusted as a competing risk event. Figure S5: cumulative incidence of ESRD in patients with liver cirrhosis before propensity score matching that was analyzed using the modified log rank test with death adjusted as a competing risk event.
