# Supplementary material for: Renal Effects of Angiotensin-Converting Enzyme Inhibitors and Angiotensin Receptor Blockers in Patients with Liver Cirrhosis: A Nationwide Cohort Study
Source: Gastroenterol Res Pract. 2019 Oct 10;2019:1743290. doi: 10.1155/2019/1743290 (PMC6811787; doi:10.1155/2019/1743290)
Supplement: Supplementary Materials — Table S1: antihypertensive agents. Table S2: ICD-9-CM codes for the diagnoses of comorbidities. Table S3: baseline characteristics of cirrhotic patients with ascites. Table S4: multivariate Cox proportional hazards model analysis of risk of ESRD in cirrhotic patients with ascites after adjustment for competing mortality. Table S5: baseline characteristics of the patients with decompensated liver cirrhosis. Table S6: baseline characteristics of patients with liver cirrhosis taking ACEi/ARB or CCB before propensity score matching. Figure S1: flowchart of the enrollment process for cirrhotic patients with ascites. ∗More than one exclusion criteria could overlap in a patient. Figure S2: cumulative incidence of ESRD in cirrhotic patients with ascites that was analyzed using the modified log-rank test with death adjusted as a competing risk event. Figure S3: flowchart of the enrollment process for patients with decompensated liver cirrhosis. ∗More than one exclusion criteria could overlap in a patient. Figure S4: cumulative incidence of ESRD in patients with decompensated liver cirrhosis that was analyzed using the modified log-rank test with death adjusted as a competing risk event. Figure S5: cumulative incidence of ESRD in patients with liver cirrhosis before propensity score matching that was analyzed using the modified log rank test with death adjusted as a competing risk event. [file 1743290.f1.pdf]

Supplementary Materials

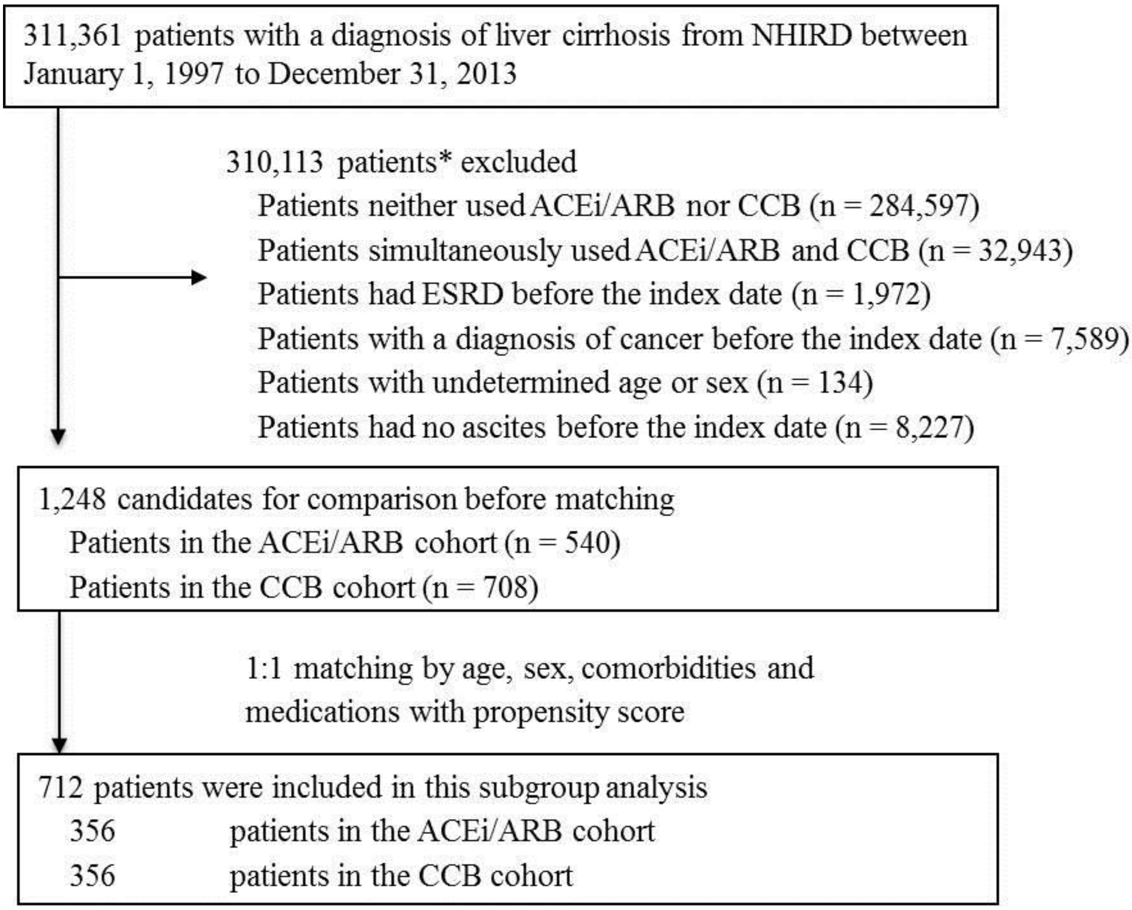

FIGURE S1: Flowchart of the enrollment process for cirrhotic patients with ascites.

*more than one exclusion criteria could overlap in a patient.

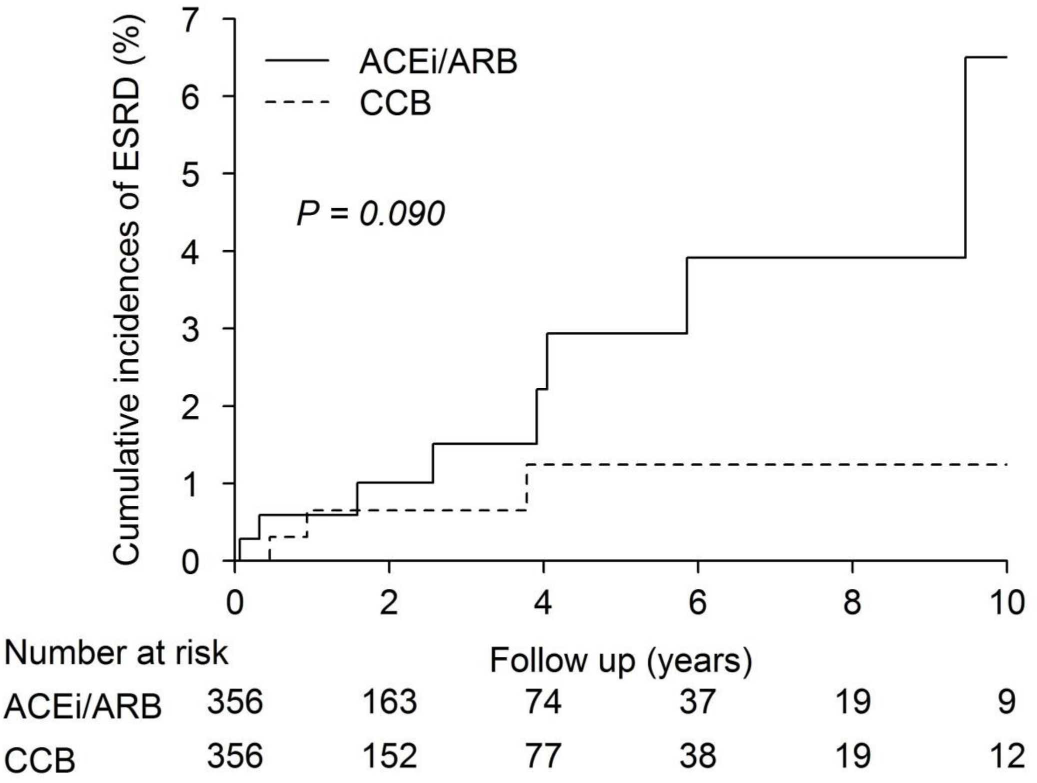

FIGURE S2: Cumulative incidence of ESRD in cirrhotic patients with ascites that was analyzed using the modified log-rank test with death adjusted as a competing risk event.

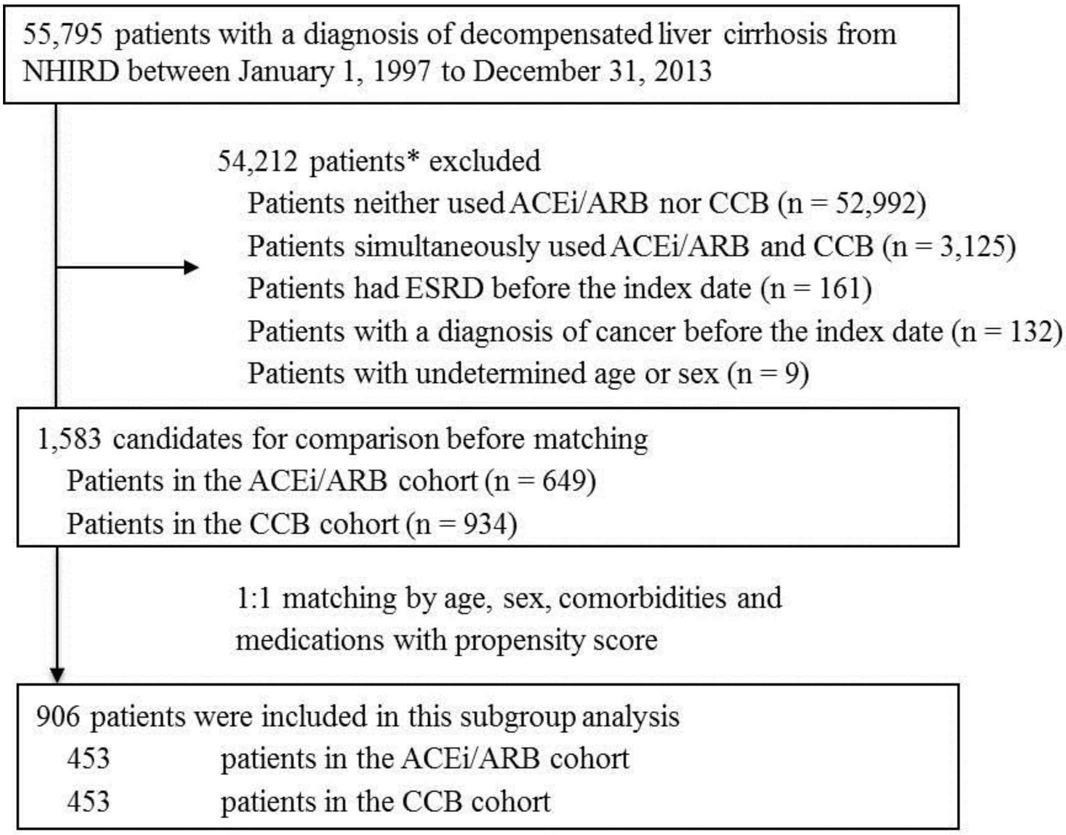

FIGURE S3: Flowchart of the enrollment process for patients with decompensated liver cirrhosis. *more than one exclusion criteria could overlap in a patient.

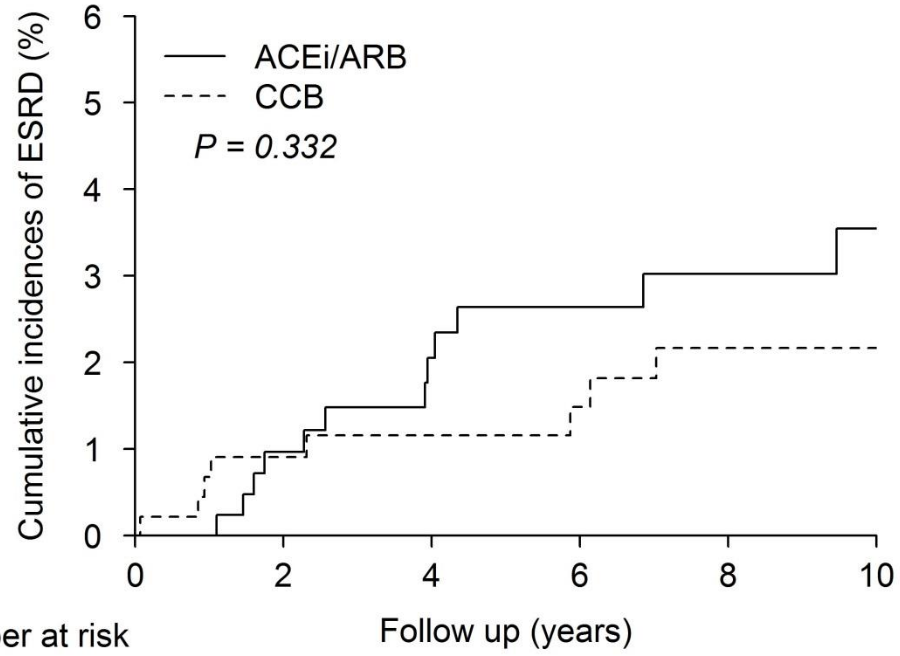

Number at risk

ACEi/ARB	453	299	185	111	50	29
CCB	453	299	177	107	54	23

FIGURE S4: Cumulative incidence of ESRD in patients with decompensated liver cirrhosis that was analyzed using the modified log-rank test with death adjusted as a competing risk event.

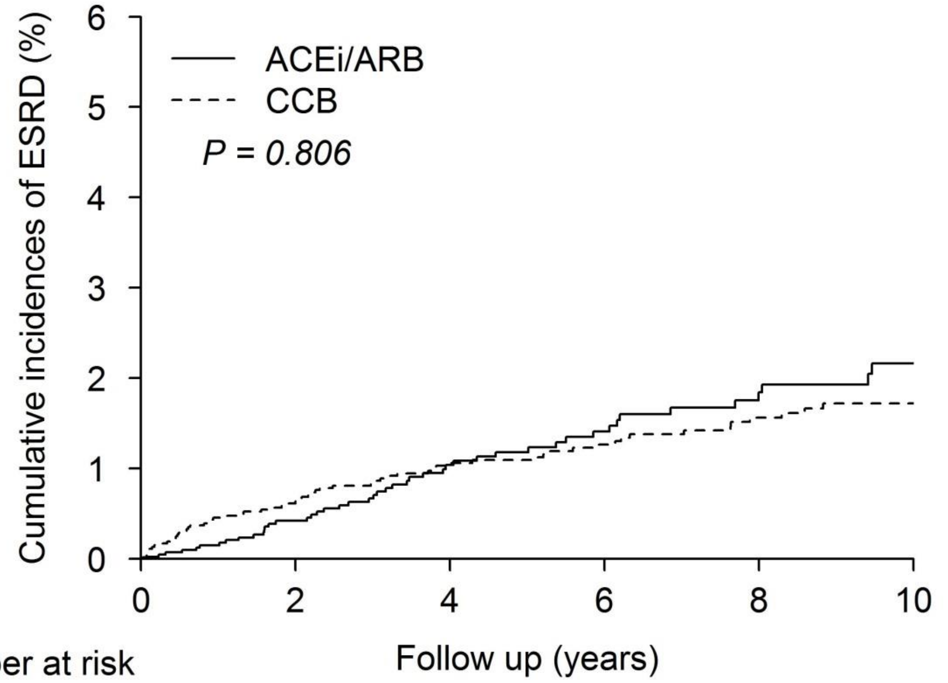

Number at risk						
ACEi/ARB	4208	2688	1625	961	541	299
CCB	5267	3528	2354	1558	964	527

FIGURE S5: Cumulative incidence of ESRD in patients with liver cirrhosis before propensity score matching that was analyzed using the modified log-rank test with death adjusted as a competing risk event.

TABLE S1: Antihypertensive agents

Drugs
Angiotensin-converting enzyme inhibitors
Benazepril
Captopril
Cilazapril
Enalapril
Fosinopril
Imidapril
Lisinopril
Perindopril
Quinapril
Ramipril
Angiotensin receptor blockers
Candesartan
Irbesartan
Losartan
Olmesartan
Telmisartan
Valsartan
Calcium channel blockers
Amlodipine
Barnidipine
Benidipine
Felodipine
Isradipine
Lacidipine
Lercanidipine
Nifedipine
Nicardipine
Nitrendipine

TABLE S2: ICD-9-CM codes for the diagnoses of comorbidities

Disease	Corresponding ICD-9-CM codes
Hepatitis B virus infection	070.2, 070.3, V02.61
Hepatitis C virus infection	070.41, 070.44, 070.51, 070.54, 070.70, 070.71, V02.62
Alcoholic liver disease	571.0-571.3
Other chronic hepatitis	571.40, 571.41, 571.49
Hypertension	401-405, A260, A269
Diabetes mellitus	249-250
Congestive heart failure	428
Hyperlipidemia	272

TABLE S3: Baseline characteristics of cirrhotic patients with ascites

Characteristics	ACEi/ARB (*n* = 356)<br>*n* (%)	CCB (*n* = 356)<br>*n* (%)	*P* value
Age, years, median (IQR)	69.93 (57.31–78.38)	69.68 (58.49–78.44)	0.891
Gender			> 0.999
Female	168 (47.2)	168 (47.2)	
Male	188 (52.8)	188 (52.8)	
Cause of cirrhosis			
Hepatitis B virus infection	50 (14.0)	55 (15.5)	0.672
Hepatitis C virus infection	39 (11.0)	33 (9.3)	0.534
Alcoholic liver disease	36 (10.1)	49 (13.8)	0.165
Other chronic hepatitis	131 (36.8)	121 (34.0)	0.481
Comorbidity			
Hypertension	302 (84.8)	290 (81.5)	0.271
Diabetes mellitus	181 (50.8)	181 (50.8)	> 0.999
Congestive heart failure	89 (25.0)	79 (22.2)	0.427
Hyperlipidemia	96 (27.0)	101 (28.4)	0.738
Drug exposure			
Beta blockers	74 (20.8)	70 (19.7)	0.780
Statins	29 (8.2)	28 (7.9)	> 0.999
Metformin	79 (22.2)	81 (22.8)	0.928
Aspirin	62 (17.4)	64 (18.0)	0.922
NSAIDs or COX2	94 (26.4)	84 (23.6)	0.436
ESRD	8 (2.25)	3 (0.84)	0.224
Competing mortality	134 (37.6)	165 (46.4)	0.023
Follow-up years (IQR)	1.67 (0.64–3.50)	1.61 (0.64–3.49)	0.748

ACEi: angiotensin-converting enzyme inhibitor; ARB: angiotensin receptor blocker; CCB: calcium channel blocker; COX-2: cyclooxygenase-2 inhibitors; ESRD: end-stage renal disease; IQR: interquartile range; NSAIDs: non-steroidal anti-inflammatory drugs.

TABLE S4: Multivariate cox proportional hazards model analysis of risk of ESRD in cirrhotic patients with ascites after adjustment for competing mortality

	HR (95% CI)	*P* value
ACEi/ARB *vs.* CCB users	3.25 (0.70–15.08)	0.132
Age	0.93 (0.90–0.97)	< 0.001
Male *vs.* female	0.24 (0.06–0.96)	0.044
Hepatitis B virus infection	0.24 (0.03–2.19)	0.204
Hepatitis C virus infection	0.77 (0.11–5.40)	0.794
Alcoholic liver disease	1.49 (0.44–5.00)	0.523
Other chronic hepatitis	–	–
Hypertension	0.55 (0.12–2.61)	0.454
Diabetes mellitus	6.66 (1.32–33.63)	0.022
Congestive heart failure	0.43 (0.06–3.07)	0.397
Hyperlipidemia	0.34 (0.06–1.98)	0.231
Beta blockers	0.41 (0.04–4.11)	0.452
Statins	–	–
Metformin	1.35 (0.43–4.28)	0.609
Aspirin	0.28 (0.03–2.70)	0.273
NSAIDs or COX2	1.17 (0.29–4.63)	0.825

ACEi: angiotensin-converting enzyme inhibitor; ARB: angiotensin receptor blocker; CCB: calcium channel blocker; CI: confidence interval; COX-2: cyclooxygenase-2 inhibitor; ESRD: end-stage renal disease; HR: hazard ratio; NSAIDs: non-steroidal anti-inflammatory drugs.

Table S5. Baseline characteristics of the patients with decompensated liver cirrhosis

	ACEi/ARB (*n* = 453)	CCB (*n* = 453)	
Characteristics	*n* (%)	*n* (%)	*P* value
Age, years, median(IQR)	65.14 (54.31–72.74)	65.23 (54.52–73.07)	.988
Gender			>.999
Female	227 (50.1)	227 (50.1)	
Male	226 (49.9)	226 (49.9)	
Cause of cirrhosis			
Hepatitis B virus infection	85 (18.8)	101 (22.3)	.217
Hepatitis C virus infection	89 (19.7)	84 (18.5)	.735
Alcoholic liver disease	60 (13.3)	72 (15.9)	.300
Other chronic hepatitis	191 (42.2)	185 (40.8)	.736
Comorbidity			
Hypertension	397 (87.6)	391 (86.3)	.622
Diabetes mellitus	256 (56.5)	240 (53.0)	.317
Congestive heart failure	63 (13.9)	66 (14.6)	.849
Hyperlipidemia	108 (23.8)	109 (24.1)	>.999
Drug exposure			
Beta blockers	81 (17.9)	89 (19.7)	.551
Statin	28 (6.2)	23 (5.1)	.564
Metformin	125 (27.6)	115 (25.4)	.498
Aspirin	57 (12.6)	53 (11.7)	.760
NSAIDs or COX2	122 (26.9)	125 (27.6)	.881
ESRD	12 (2.65)	8 (1.77)	.498
Competing mortality	286 (63.1)	313 (69.1)	.068
Follow-up year (IQR)	3.19 (1.41–5.99)	2.92 (1.32–5.77)	0.707

ACEi: angiotensin converting enzyme inhibitor; ARB: angiotensin receptor blocker; CCB: calcium channel blocker; COX-2: cyclooxygenase-2 inhibitor; ESRD: end stage renal disease; IQR: interquartile range; NSAIDs: non-steroidal anti-inflammatory drugs.

TABLE S6: Baseline characteristics of patients with liver cirrhosis taking ACEi/ARB or CCB before propensity score matching

Characteristics	ACEi/ARB (*n* = 4,208) *n* (%)	CCB (*n* = 5,267) *n* (%)	*P* value
Age, years, median (IQR)	66.41 (56.29–75.08)	67.55 (58.45–75.20)	< 0.001
Gender			< 0.001
Female	1590 (37.8)	2334 (44.3)	
Male	2618 (62.2)	2933 (55.7)	
Cause of cirrhosis			
Hepatitis B virus infection	664 (15.8)	684 (13.0)	< 0.001
Hepatitis C virus infection	666 (15.8)	890 (16.9)	0.171
Alcoholic liver disease	351 (8.3)	416 (7.9)	0.455
Other chronic hepatitis	1833 (43.6)	2243 (42.6)	0.352
Comorbidity			
Hypertension	3576 (85.0)	4801 (91.2)	< 0.001
Diabetes mellitus	2015 (47.9)	1737 (33.0)	< 0.001
Congestive heart failure	1025 (24.4)	433 (8.2)	< 0.001
Hyperlipidemia	1337 (31.8)	1133 (21.5)	< 0.001
Drug exposure			
Beta blockers	1334 (31.7)	1497 (28.4)	< 0.001
Statins	742 (17.6)	486 (9.2)	< 0.001
Metformin	1402 (33.3)	1201 (22.8)	< 0.001
Aspirin	1265 (30.1)	1168 (22.2)	< 0.001
NSAIDs or COX2	1680 (39.9)	2314 (43.9)	< 0.001
ESRD	47 (1.12)	65 (1.23)	0.668
Competing mortality	1321 (31.4)	2138 (40.6)	< 0.001
Follow-up year (IQR)	2.99 (1.31–5.69)	3.53 (1.47–6.83)	< 0.001

ACEi: angiotensin-converting enzyme inhibitor; ARB: angiotensin receptor blocker; CCB: calcium channel blocker; COX-2: cyclooxygenase-2 inhibitor; ESRD: end-stage renal disease; IQR: interquartile range; NSAIDs: non-steroidal anti-inflammatory drugs.
